# Integrated single-cell transcriptomic analysis identifies *PF4*^*+*^*/PPBP*^*+*^ megakaryocyte-like granulocytes associated with immune dysregulation in autoimmune diseases

**DOI:** 10.1016/j.bbrep.2026.102615

**Published:** 2026-05-10

**Authors:** Shaoqi Chen, Yu Fan, Miaotong Su, Yuqing Lin, Shaoyu Zheng, Zexuan Zhou, Weijin Zhang, Jianqun Lin, Shijian Hu, Marco Matucci-Cerinic, Daniel E. Furst, Guohong Zhang, Yukai Wang

**Affiliations:** a Department of Ultrasound, The First Affiliated Hospital of Shantou University Medical College, Shantou, Guangdong, China; b Department of Pathology, Shantou University Medical College, Shantou, China; c Department of Rheumatology and Immunology, Shantou Central Hospital, Shantou, China; d Unit of Immunology, Rheumatology, Allergy and Rare Diseases (UnIRAR), and Inflammation, Fibrosis and Ageing Initiative (INFLAGE), IRCCS San Raffaele Hospital, Milano, Italy; e University of California Los Angeles, Los Angeles, USA

**Keywords:** Megakaryocytes, Low-density granulocytes, Autoimmune disease, NET formation, Cell communication

## Abstract

**Objectives:**

Megakaryocytes (MKs) and low-density granulocytes (LDGs) are implicated in immune dysregulation and vascular pathology in autoimmune diseases (ADs), yet their precise subsets and pathological interactions remain poorly defined. We aimed to characterize MK and LDG subpopulations and elucidate their potential intercellular communication in ADs using single-cell transcriptomic analysis.

**Methods:**

Single-cell RNA sequencing (scRNA-seq) was performed on peripheral blood mononuclear cells from 10 treatment-naive AD patients (4 pSS, 3 RA, and 3 SLE) and 3 healthy controls (HCs). MKs and LDGs were re-clustered to identify transcriptional subpopulations and interrogated for intercellular communication using CellChat. A distinct megakaryocyte-like granulocyte population was validated in an independent scRNA-seq dataset. Bulk RNA-seq (n = 139) and plasma ELISA assays were employed to support the associated molecular signatures. Crucially, flow cytometry of peripheral blood from AD patients (n = 5) and HCs (n = 4) was performed to provide protein-level validation of the identified megakaryocyte-like granulocytes.

**Results:**

MKs segregated into immune-active and platelet-generating subtypes, both exhibiting altered signaling in ADs. LDGs harbored a unique *PF4*^*+*^*/PPBP*^*+*^ megakaryocyte-like subpopulation with heightened interferon activity, proinflammatory signaling, and transcriptional signatures of increased neutrophil extracellular trap (NET) formation. Flow cytometry confirmed the presence of these granulocytes and showed a higher proportion in AD patients than in HCs. These granulocytes showed predicted communication with MKs via ITGB2-ICAM2 and APP-CD74 axes. Findings were consistently validated in an external scRNA-seq dataset and corroborated by bulk RNA-seq deconvolution and elevated plasma myeloperoxidase levels.

**Conclusions:**

We identify a potentially *PF4*^*+*^*/PPBP*^*+*^ megakaryocyte-like granulocyte subset associated with immune dysregulation in ADs. While flow cytometry provides protein-level evidence for this population, further mechanistic studies are required to fully elucidate its functional role in disease pathogenesis.

## Highlights

• Single-cell transcriptomics identifies megakaryocyte-like LDGs in autoimmune diseases.
• Megakaryocyte-like LDGs exhibit signatures of inflammation and NET formation.
• Aberrant cell-cell crosstalk via ITGB2-ICAM2 and APP-CD74 drives immune dysregulation.

## Graphical abstract

**Figure:**
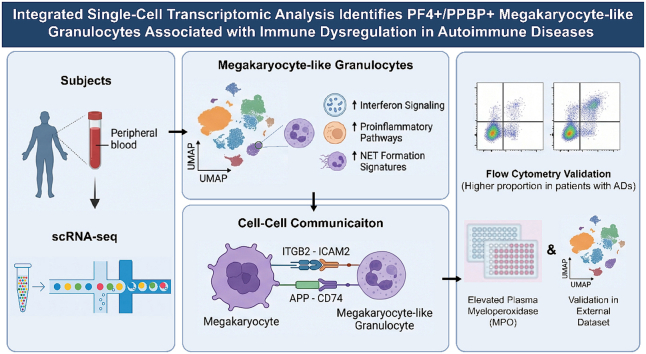

## 1 Introduction

Autoimmune diseases (ADs) affect approximately 3.2% of the global population [1], and include rheumatoid arthritis (RA), primary Sjögren's syndrome (pSS), and systemic lupus erythematosus (SLE) as the most common forms [[2], [3], [4]]. These disorders share key immunological features, including dysregulated type I interferon signaling, chronic inflammation, and the production of autoantibodies, often linked to neutrophil extracellular trap (NET) formation [5]. While these processes provide a framework for humoral autoimmunity, the cellular mechanisms sustaining chronic inflammation remain incompletely defined.

Megakaryocytes (MKs), traditionally regarded as platelet precursors, are now recognized as immune-active cells with antigen-presenting and cytokine-secreting functions [[6], [7], [8]]. We previously reported an expansion of immune-type MKs in RA, pSS, and SLE, suggesting their contribution to immune dysregulation [9]. Low-density granulocytes (LDGs) are a proinflammatory neutrophil subset characterized by exaggerated NETosis and altered transcriptional programs that promote tissue damage and autoantibody production in autoimmune diseases [10]. Despite their individual relevance, potential interactions between MKs and LDGs in ADs have not been systematically explored.

A possible link is emperipolesis, in which neutrophils transiently enter MKs, a process dependent on integrin signaling and cytoskeletal remodeling [11,12]. Unlike phagocytosis, both the MK and the LDG remain viable and functionally active after the interaction. Although implicated in platelet biogenesis and immune modulation [13], the molecular basis and relevance of this interaction in autoimmunity remain unclear.

Here, using single-cell RNA sequencing (scRNA-seq), we identified a distinct LDG subpopulation expressing megakaryocyte-associated genes (PF4, PPBP), termed megakaryocyte-like LDGs (MK-LDGs). Crucially, these cells represent a subset of LDGs that have aberrantly acquired specific megakaryocyte/platelet-associated signatures rather than being bona fide megakaryocytes. These cells retain neutrophilic identity but display features of platelet-producing lineages, likely reflecting transcriptional reprogramming within the inflammatory milieu rather than direct cell-in-cell interactions or true lineage *trans*-differentiation. Our findings reveal a previously unrecognized MK–LDG axis in ADs and suggest new mechanisms of innate immune dysregulation that are amenable to therapeutic targeting.

## 2 Methods

### 2.1 Patient selection

Peripheral blood samples for single-cell sequencing were collected from treatment-naive patients, including 3 with RA, 3 with SLE, and 4 with pSS, as well as from 3 healthy controls (HCs) matched for sex, ethnicity, and age, all of whom were recruited at Shantou Central Hospital (Guangdong, China). Detailed demographic and clinical characteristics of all participants, including age, gender, and statistical comparisons, are summarized in Supplementary Table 1. Bulk RNA-seq data were obtained from our database, comprising 119 treatment-naive patients with autoimmune diseases (48 with RA, 38 with pSS, and 33 with SLE) and 20 healthy controls. In addition, plasma samples were collected from 31 of the 119 patients with ADs and 10 healthy controls for ELISA analysis. All the subjects provided written informed consent. Patients met the following diagnostic criteria: the 2002 American–European Consensus Group criteria for pSS, the 2019 EULAR/ACR Classification Criteria for SLE, and the 2010 ACR/EULAR criteria for RA. The inclusion criteria for AD patients were a confirmed diagnosis according to these international criteria, being treatment-naive at the time of sampling, and the absence of concurrent infections or malignancies. Healthy controls (HCs) were individuals with no history of autoimmune disease, inflammatory disorders, or malignancies, and were not receiving immunosuppressive therapy.

### 2.2 scRNA-seq and data processing

Single cells were sequenced on the 10 × Genomics platform. PBMCs were isolated from undiluted human blood via Histopaque (Cat.#: 10771; Sigma-Aldrich, St. Louis, MO, USA). Barcoded scRNA-seq libraries were generated via Chromium Single cell 3′ Reagent v3 kits, following the manufacturer's protocol. Raw data were processed with Cell Ranger (version 3.1.0) for demultiplexing and FASTQ generation, followed by alignment to the human reference genome (GrCh38). The resulting UMI count matrix was converted into a Seurat object (version 5.0.1) [14]. Cells with doublets or low quality were removed based on UMI counts and mitochondrial gene expression (threshold >20%). Following log-normalization and selection of the top 2000 highly variable genes, principal component analysis (PCA) was performed for dimensionality reduction. Based on the Elbow plot, the first 30 principal components (PCs) were selected for further dimensionality reduction via UMAP and neighbor identification. Cell clusters were identified using the FindNeighbors (based on the 30 PCs) and FindClusters functions with a resolution parameter of 0.2. Batch correction was conducted using Harmony to ensure data consistency across samples.

### 2.3 Identification of functional cellular subsets within major cell clusters

Functional cellular subsets were identified using Seurat's FindAllMarkers function (Wilcoxon test). Marker genes were ranked by log fold change (logFC), and cell subtypes were defined based on the expression of established canonical markers.

### 2.4 Gene set variation analysis (GSVA) for functional identification of cell subclusters

GSVA was performed to identify functional pathways using the GOBP database retrieved via the msigdbr package (version 7.5.1). Pathway activity estimates were assigned to individual cells via the GSVA package (version 1.50.0) [15].

### 2.5 Differential expression and functional enrichment analysis across cell types and disease states

Differentially expressed genes (DEGs) were identified across various cell types and disease states using Seurat's FindMarkers function (logFC threshold = 0.25, adjusted *p*-value <0.05) via the Wilcoxon test. o ensure robust identification, DEGs were defined as genes with an adjusted *p*-value <0.05 and |logFC| > 1. The tissue and cell-type specificity of these DEGs was inferred using the WebCSEA tool (https://bioinfo.uth.edu/webcsea/) [16]. To investigate biological functions and pathways, Gene Ontology Biological Process (GOBP) [17] and Kyoto Encyclopedia of Genes and Genomes (KEGG) [18] pathway enrichment analyses were conducted using Metascape (https://metascape.org/) [19]. Visualization of the enrichment results was performed using the ggplot2 package (version 3.5.1) in R.

### 2.6 Calculation of multiple identity scores for LDGs

To assess the transcriptional the identity of the LDGs, peripheral blood, cord blood, and bone marrow samples from the human cell landscape [20] database (GSE134355) were utilized as reference single-cell datasets. Multiple identity scores for LDGs were calculated using the “multi.id.curate.qp” function from the Capybara package (version 0.0.0.9000) [21] to evaluate potential lineage overlap.

### 2.7 Cell-cell interaction analysis

Cell-cell interactions between different cell types were evaluated using the CellChat R package (version 1.6.0). This tool estimates the communication probability by integrating gene expression data with a curated database of known interactions involving ligands, receptors, and cofactors [22]. Normalized counts from each condition were used to generate CellChat objects, and the recommended preprocessing steps were applied with default parameters for both individual and comparative dataset analysis.

### 2.8 Validation cohort

To validate our results, we analyzed scRNA-seq datasets of PBMCs from AD patients obtained from Gene Expression Omnibus (GEO, https://www.ncbi.nlm.nih.gov/geo/). The datasets included GSE142016, comprising three SLE samples (GSM4217718 to GSM4217720) [22], GSE157278, with five pSS samples (GSM4760625 to GSM4760629) [23], GSE159117, containing one RA sample (GSM4819747) [24], GSE162577, with two SLE samples (GSM4954811, and GSM4954812) [25], GSE224198, including four SLE samples (GSM7017326, GSM7017329, GSM7017331, and GSM7017334) [26], and GSE263931, consisting of seven SLE samples (GSM8207595, GSM8207597, GSM8207599, GSM8207601, GSM8207603, GSM8207605, and GSM8207607) [27]. The analytical methods employed were consistent with those used for the discovery cohort.

### 2.9 Gene set enrichment analysis in the bulk RNA-seq cohort

To evaluate neutrophil extracellular trap (NET) formation signatures in the bulk RNA-seq cohort, GSEA was performed using the "Neutrophil Extracellular Trap Formation" pathway (hsa04613) from KEGG. Analysis and visualization were conducted utilizing the clusterProfiler (version 4.8.3) and enrichplot (version 1.20.3) packages, respectively.

### 2.10 BayesPrism deconvolution analysis of bulk RNA-seq

Bulk RNA-seq data were deconvoluted into constituent cell types using the BayesPrism R package (version 2.2.2). BayesPrism utilizes a probabilistic framework to infer cell type-specific gene expression from bulk data guided by scRNA-seq references [28]. Deconvolution was performed using 200 randomly sampled cells per cell type as a reference with default parameters.

### 2.11 Enzyme-linked immunosorbent assay (ELISA) for detecting neutrophil elastase (NE)

Given its role as a key biomarker for NET formation, plasma neutrophil elastase (NE) levels were measured using the Human Neutrophil Elastase ELISA Kit (Cat.#: ab270204, Abcam, Cambridge, UK). Assays were carried out according to the manufacturer's protocol with plasma samples diluted 1:100. We analyzed 31 AD patients selected from the bulk RNA-seq cohort and 10 healthy controls. The correlation between NE levels and deconvolution-derived MK-LDG scores was assessed using Spearman correlation.

### 2.12 Flow cytometry validation of MK-LDGs

To ascertain the protein-level existence of MK-LDGs, peripheral blood samples were collected from an independent validation cohort comprising five treatment-naive AD patients (stratified as 1 RA, 2 SLE, and 2 pSS) and three age- and sex-matched HCs. PBMCs were isolated via Histopaque-1077 density gradient centrifugation. Subsequently, cells were stained with fluorochrome-conjugated antibodies against CD16b (granulocyte/LDG marker; Cat.#: 555406, BD Biosciences, USA) and PF4 (megakaryocyte marker; Cat.#: 170138, R&D Systems, UK).Appropriate isotype controls were employed to define gating boundaries and account for non-specific binding. Data acquisition was performed using standard flow cytometry protocols, and results were analyzed using FlowJo software (v10.8.1). The frequency of CD16b^+^PF4^+^ cells was quantified as a percentage of total PBMCs.

### 2.13 Statistical analyses

Statistical analyses were conducted using R software (version 4.3.2). Inter-group comparisons were evaluated using the Kruskal-Wallis and Wilcoxon rank-sum tests, while associations were determined via Spearman correlation. All statistical tests were two-tailed, and significance was defined as *p*-value <0.05.

## 3 Results

### 3.1 Expansion and functional reprogramming of megakaryocytes (MKs) and low-density granulocytes (LDGs) in autoimmune disease

**Fig. 1:**
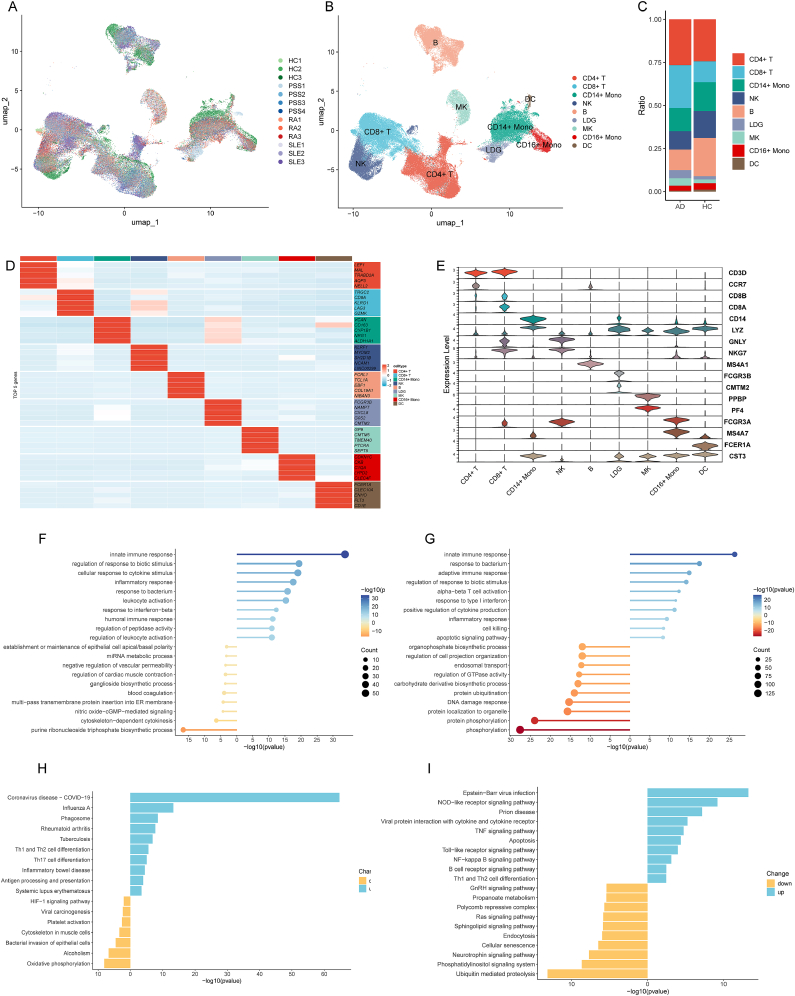
**Characterization of PBMCs and Functional Enrichment in MKs and LDGs.** (A) UMAP plot of scRNA-seq data from 114,848 PBMCs across AD and HC groups. (B) UMAP annotated with identified cell types. (C) Cell-type proportions in AD vs. HC. (D) Heatmap of top five marker genes per cluster. (E) Violin plots showing canonical marker gene expression across clusters. (F) Gene Ontology Biological Process (GOBP) enrichment of DEGs in MKs. (G) GOBP enrichment of DEGs in LDGs. (H) KEGG pathway enrichment of DEGs in MKs. (I) KEGG pathway enrichment of DEGs in LDGs. Left bars: downregulated pathways; right bars: upregulated pathways. AD, autoimmune disease; HCs, healthy controls; pSS, primary Sjögren's syndrome; RA, rheumatoid arthritis; SLE, systemic lupus erythematosus.

Following stringent quality control, scRNA-seq analysis of 114,848 PBMCs from 10 AD patients and 3 HCs (Fig. 1A and B) identified nine major immune cell populations based on canonical markers [[29], [30], [31]] (Fig. 1D–E): CD4^+^ T cells, CD8^+^ T cells, CD14^+^ monocytes, natural killer (NK) cells, B cells, LDGs, MKs, CD16^+^ monocytes, and dendritic cells. The UMAP projections for HC, RA, SLE, and pSS cohorts are separately illustrated in Supplementary Figure 1. Notably, MKs and LDGs were significantly enriched in AD samples compared to HCs (Fig. 1C).

Functional enrichment of differentially expressed genes (DEGs) revealed that AD-derived MKs underwent a shift from a thrombopoietic to a proinflammatory phenotype, characterized by the upregulation of innate immune and inflammatory pathways (Fig. 1F and H) and the downregulation of platelet activation and maturation genes. Similarly, LDGs in AD patients exhibited functional reprogramming, with marked activation of TNF, NF-κB, and toll-like receptor signaling (Fig. 1G and I). Conversely, pathways involved in protein phosphorylation and ubiquitin-mediated proteolysis were downregulated. Collectively, these findings indicate that the expansion and immune activation of MKs and LDGs are shared immunopathological features that drive systemic immune dysregulation across ADs.

### 3.2 Functional characterization of MK subpopulations in autoimmune disease

**Fig. 2:**
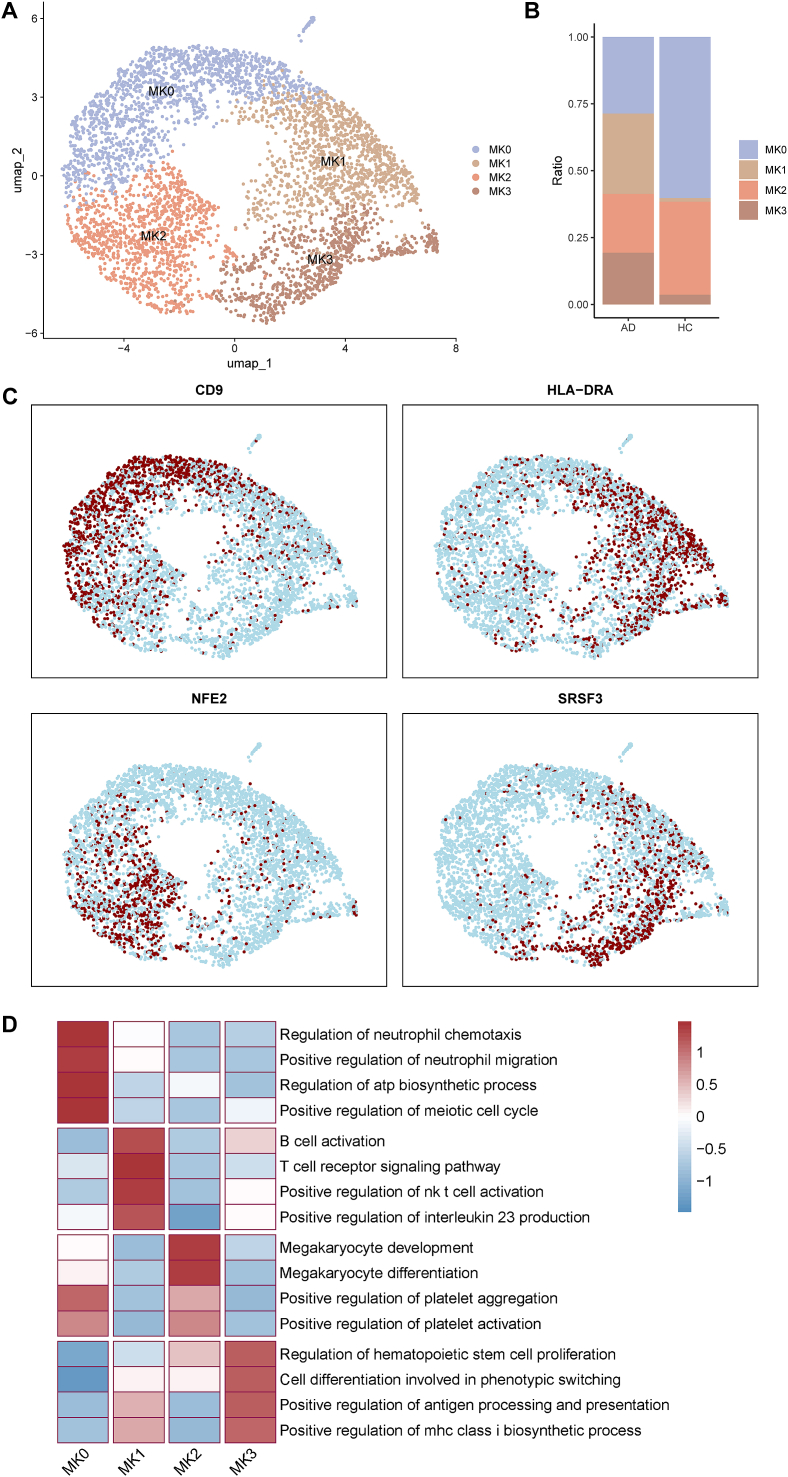
**MK Subpopulations and Their Functional Signatures in Autoimmune Disease.** (A) UMAP visualization of reclustered MKs showing four subpopulations: MK0, MK1, MK2, and MK3. (B) Proportions of each MK subset in healthy controls (HCs) and autoimmune disease (AD) patients. (C) Expression levels of representative marker genes (CD9, HLA-DRA, NFE2, SRSF3) across MK subpopulations. (D) Gene set variation analysis (GSVA) showing functional enrichment scores for hallmark biological processes in each MK subpopulation. Red: upregulated; Blue: downregulated; color intensity reflects enrichment magnitude. MK, megakaryocyte; AD, autoimmune disease; HC, healthy control.

To characterize megakaryocyte (MK) heterogeneity in autoimmune diseases (AD), MKs were subsetted and re-clustered, identifying four transcriptionally distinct subpopulations designated MK0–MK3 (Fig. 2A). Notably, MK1 and MK3 were significantly expanded in AD patients relative to healthy controls (HCs) (Fig. 2B), indicating a disease-associated shift in MK subset composition. The subpopulations were defined by specific marker genes: *CD9* (MK0), *HLA-DRA* (MK1), *NFE2* (MK2), and *SRSF3* (MK3) (Fig. 2C). These markers reflect a functional spectrum within the MK lineage, spanning from classical thrombopoietic to immune-active states. Gene set variation analysis (GSVA) further delineated their distinct functional profiles (Fig. 2D). MK0 was enriched for neutrophil chemotaxis and migration, suggesting a role in myeloid cell recruitment. MK1 exhibited strong signatures of T cell receptor signaling and NK cell activation, indicating involvement in lymphocyte–MK crosstalk. MK2 maintained a classical thrombopoietic phenotype, characterized by genes related to megakaryocyte development and platelet function. In contrast, MK3 was primarily associated with hematopoietic stem cell proliferation and inflammatory reprogramming.

Importantly, MK1 and MK3 co-enriched for antigen processing, MHC class I biosynthesis, and interferon signaling, suggesting potential antigen-presenting and immunoregulatory functions. Conversely, MK0 and MK2 showed downregulation of platelet-related signatures, further distinguishing immune-oriented from hemostatic MK subsets. Collectively, these findings demonstrate a transition toward immune-competent MK subsets in AD, which likely facilitate antigen presentation and contribute to systemic immune dysregulation [9].

### 3.3 Functional characterization of LDG subpopulations in autoimmune disease

**Fig. 3:**
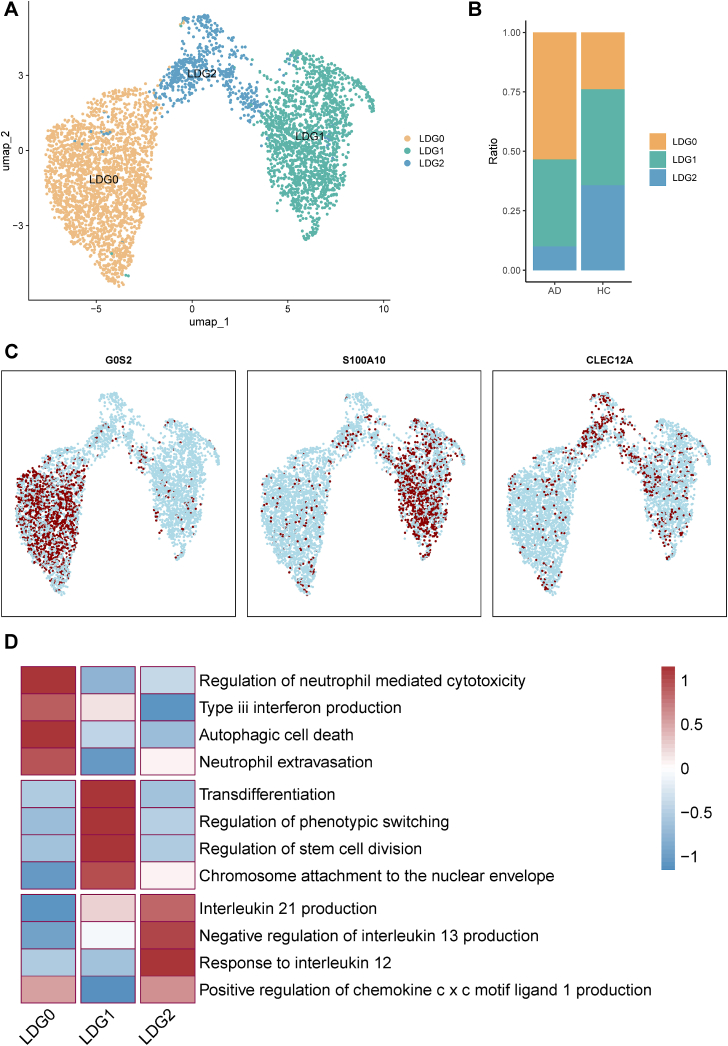
LDG Subpopulations and Their Functional Signatures in Autoimmune Disease. (A) UMAP visualization of LDG subpopulations, highlighting LDG0, LDG1, and LDG2. (B) Comparison of LDG subpopulation proportions between healthy controls (HC) and autoimmune disease (AD) patients. (C) Expression of representative marker genes (G0S2, S100A10, CLEC12A) across LDG subsets. (D) Gene ontology enrichment profiles for each LDG subset, as assessed by GSVA. Red: positive enrichment; Blue: negative enrichment; color intensity reflects magnitude. LDG, low-density granulocyte; HC, healthy control; AD, autoimmune disease.

To delineate the functional heterogeneity of low-density granulocytes (LDGs) and investigate the potential emergence of megakaryocyte-like features in autoimmune disease (AD), we conducted unsupervised clustering analysis. This identified three transcriptionally distinct LDG subpopulations: LDG0, LDG1, and LDG2 (Fig. 3A). Among them, LDG0 was significantly expanded in AD patients compared to healthy controls (HCs) (Fig. 3B), suggesting disease-specific enrichment of this subset. Subcluster-specific marker genes were identified, with LDG0 expressing *G0S2*, LDG1 expressing *S100A10*, and LDG2 expressing *CLEC12A* (Fig. 3C).

Gene Ontology (GO) enrichment analysis revealed distinct functional specializations across these subsets (Fig. 3D). LDG0 showed strong enrichment for pathways associated with neutrophil-mediated cytotoxicity, degranulation, and extravasation, reflecting a hyperactivated effector phenotype that may exacerbate tissue inflammation in AD. LDG1 exhibited signatures of cellular transdifferentiation, stress adaptation, and phenotypic switching, pointing toward a plastic state potentially responsive to inflammatory cues. LDG2 was characterized by interleukin-related signaling, including IL-12 response and IL-21 production, highlighting a cytokine-modulatory role within the immune network.

While this analysis reveals distinct LDG subsets, it did not directly identify a population exhibiting canonical megakaryocyte (MK) features. Consequently, although emperipolesis-mediated MK-LDG interactions remain a compelling hypothesis, our clustering results suggest that such events may yield transcriptionally distinct states not captured by standard lineage markers alone. Further integrative analysis is required to specifically assess the presence of MK-like LDGs and their potential role in immune dysregulation.

### 3.4 Identification of MK-associated LDGs with immune- and platelet-related functions in AD

**Fig. 4:**
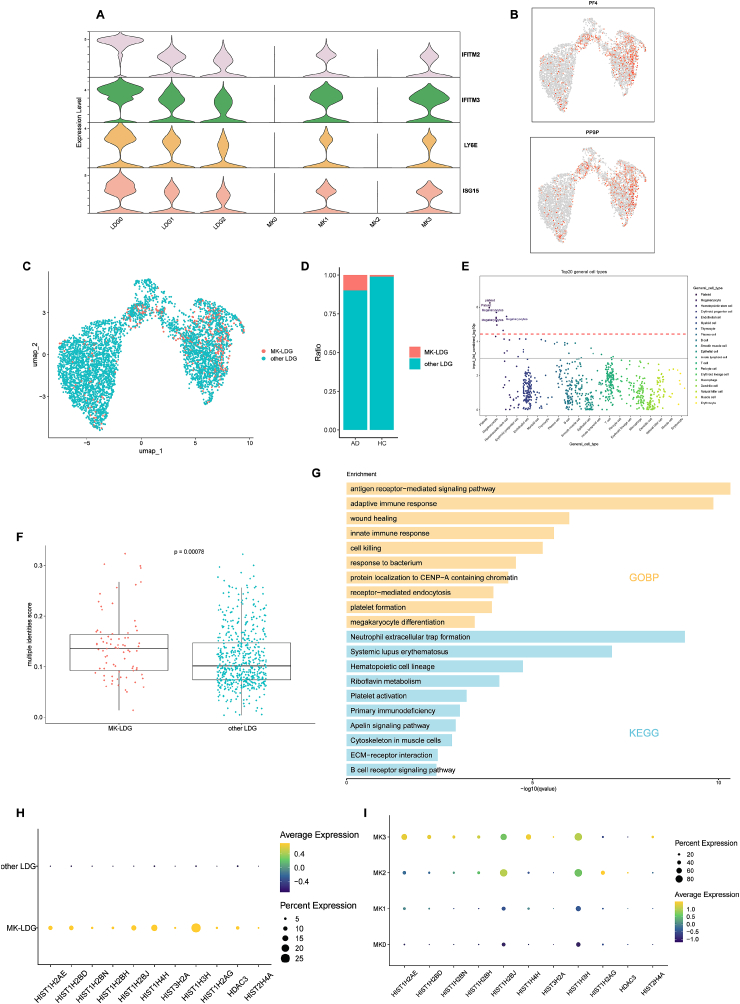
**Identification and Characterization of MK-LDGs in Autoimmune Disease.** (A) Violin plots showing the expression levels of interferon-related genes (*IFITM2, IFITM3, LY6E, and ISG15*) across the LDG and MK subclusters. (B) Feature plots displaying expression of MK markers *PF4* and *PPBP* in LDG clusters. (C) UMAP highlighting MK-LDGs (PF4/PPBP-positive LDGs) versus other LDGs. (D) Proportion of MK-LDGs versus other LDGs in AD and HC groups. (E) WebCSEA results showing top 20 enriched reference cell types for MK-LDGs, highlighting platelet/MK identities. (F) Comparison of multiple identity scores between MK-LDGs and other LDGs (Wilcoxon test). (G) GO and KEGG pathway enrichment for genes upregulated in MK-LDGs. (H, I) Dot plots showing expression of histone-related genes in MK-LDGs (H) and MK subsets (I).

Since initial unsupervised clustering did not resolve a distinct population with megakaryocyte (MK)-like features, we conducted targeted analyses to identify LDGs exhibiting MK-specific transcriptional signatures. To assess the functional overlap between LDGs and MKs, we evaluated the expression of interferon-stimulated genes (ISGs)—such as IFITM2, IFITM3, LY6E, and ISG15—which are established defining features of LDGs in systemic autoimmune diseases [32]. Notably, these ISGs were also robustly expressed in MK subpopulations MK1 and MK3, suggesting a shared interferon-driven activation program between these two cell types (Fig. 4A).

To identify LDGs with MK-associated features, we profiled the expression of canonical MK markers PF4 and PPBP within the LDG compartment. This revealed a population of MK-marker-expressing LDGs, hereafter referred to as MK-like LDGs (MK-LDGs), distributed across all three LDG subclusters (Fig. 4B and C). Importantly, MK-LDGs were significantly more abundant in AD patients than in healthy controls, suggesting a disease-associated expansion of this hybrid population (Fig. 4D).

Transcriptomic comparison between MK-LDGs and other LDGs identified a set of upregulated genes (log_2_FC > 1.5, adjusted p < 0.05) enriched for platelet and MK-associated functions, as confirmed by WebCSEA analysis (Fig. 4E). MK-LDGs also exhibited significantly elevated multiple identity scores (p = 0.00078), indicating co-expression of transcriptional programs from distinct lineages (Fig. 4F), possibly reflecting cross-lineage signaling, transcriptional plasticity, or previously unrecognized interactions between LDGs and MKs.

Functional enrichment of MK-LDG-specific genes revealed involvement in innate and adaptive immune responses, platelet activation, and MK differentiation. KEGG analysis further showed enrichment in neutrophil extracellular trap (NET) formation, systemic lupus erythematosus (SLE), hematopoietic lineage commitment, and platelet degranulation (Fig. 4G).

Consistent with their role in NET formation, MK-LDGs exhibited elevated expression of histone-related genes, such as HIST1H2BJ and HIST1H3H (Fig. 4H)—a feature also observed in the MK2 and MK3 subpopulations (Fig. 4I). This suggests that MK-LDGs are primed for chromatin-based immune responses and further supports the hypothesis that they represent a transcriptionally hybrid, functionally dysregulated population within the autoimmune milieu.

In summary, these findings identify MK-LDGs as a previously unrecognized, AD-enriched population combining transcriptional features of megakaryocytes and activated LDGs. Their functional profile suggests a role in immune dysregulation, platelet-like effector functions, and NET-driven inflammation, providing new insight into granulocyte heterogeneity in autoimmune pathogenesis.

### 3.5 MK-LDGs exhibit unique communication patterns with MKs

To further understand the role of MK-LDGs in autoimmune disease (AD), we explored their intercellular communication landscape, particularly in relation to megakaryocytes (MKs). Using CellChat, we systematically assessed ligand-receptor-mediated signaling among MKs, MK-LDGs, and other immune populations.

**Fig. 5:**
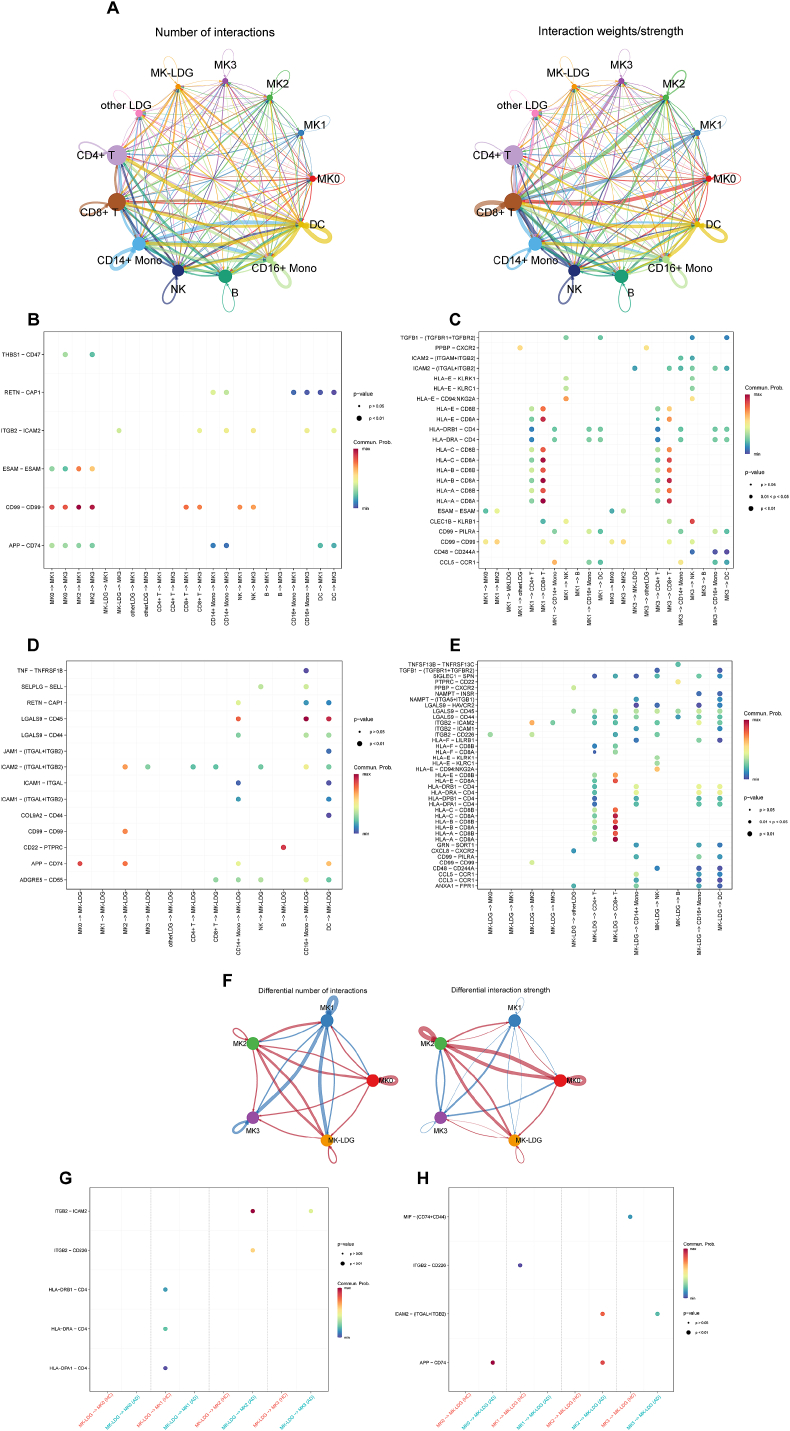
**Cell-Cell Communication Between MKs, MK-LDGs, and Other Immune Cells.** (A) Network plots illustrating the overall number and strength of interactions between various cell types, with line thickness indicating the intensity of interactions. (B) Dot plot showing signals from other cell types to MK1/MK3. (C) Signals from MK1/MK3 to other cell types. (D) Signals from other cell types to MK-LDGs. (E) Signals from MK-LDGs to other cell types. (F) Comparative network plots showing differences in interaction numbers and strengths between AD patients and HCs, with red lines indicating stronger or more numerous interactions in AD patients and blue lines indicating stronger or more numerous interactions in HCs. (G) Dot plot displaying differences in signaling from MK-LDGs to MKs between AD patients and HCs. (H) Differences in signaling from MKs to MK-LDGs between AD patients and HCs.

The global interaction network revealed that MK1, MK3, and MK-LDGs are highly interactive cell types, engaging in widespread communication with both innate and adaptive immune cells. Notably, they participated in antigen presentation signaling, including HLA-A/CD8A and HLA-DRA/CD4 axes, indicating their potential role in T cell activation and immune modulation (Fig. 5A–C). Focusing on MK-LDG–MK crosstalk, we identified several specific signaling pathways that distinguished these interactions. For instance, ICAM2 expressed on MKs interacted with ITGAL/ITGB2 (LFA-1) on MK-LDGs—molecules involved in adhesion and immune synapse formation—while PPBP (CXCL7) on MKs selectively engaged CXCR2 on non-MK LDGs, suggesting differential chemokine-mediated recruitment or activation mechanisms (Fig. 5D and E).

Comparative analysis of communication networks between AD patients and healthy controls (HCs) revealed substantial remodeling of signaling pathways. AD samples exhibited enhanced communication between MKs and MK-LDGs, with increased signaling strength and frequency (Fig. 5F). Specifically, MK-LDGs in AD showed upregulated signaling toward MKs via ITGB2-ICAM2 and ITGB2-CD226 interactions—pathways associated with immune cell adhesion and activation (Fig. 5G), Conversely, MKs in AD patients exhibited increased outgoing signals toward MK-LDGs through ICAM2-(ITGAL + ITGB2) and APP-CD74 axes (Fig. 5H), the latter implicating APP-mediated inflammatory signaling and CD74-associated antigen processing in disease pathology.

Strikingly, MK subpopulations with elevated histone gene expression—MK2 and MK3—exhibited stronger interactions with MK-LDGs compared to MK1, suggesting that chromatin remodeling and NET-associated features may facilitate heightened immune engagement in AD.

Taken together, these findings delineate a specialized, disease-associated communication network between MKs and MK-LDGs in AD, characterized by augmented adhesion, pro-inflammatory signaling, and potential pathways related to cell-in-cell interactions. These enhanced interactions likely amplify immune dysregulation and contribute to the persistent inflammatory state observed in autoimmune conditions.

### 3.6 Validation and functional characterization of MK-LDGs in an external cohort

To reinforce the robustness and generalizability of our findings, we validated the presence and functional properties of MK-LDGs using an independent single-cell dataset from the GEO database. This external cohort included 195,373 peripheral blood mononuclear cells (PBMCs) from five patients with pSS, one with RA, and 16 with SLE, providing a comprehensive spectrum of autoimmune diseases (ADs).

**Fig. 6:**
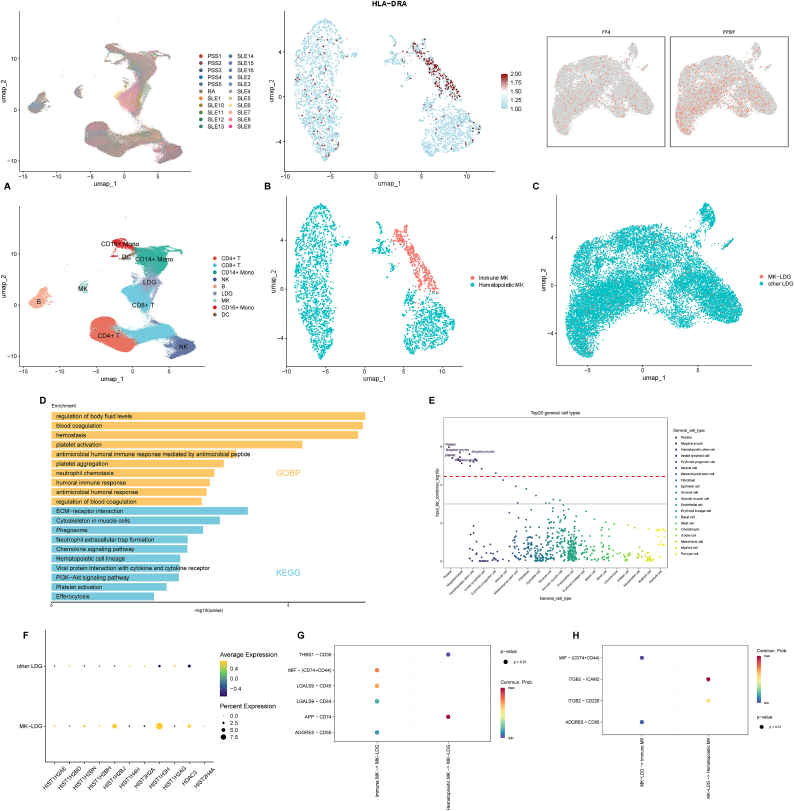
**MK-LDG Associations in the External Validation Cohort.** (A) UMAP plot displaying single-cell clusters colored by disease type (top) and annotated cell types (bottom). (B) Expression of marker genes for immune MKs (top) and UMAP visualization of immune versus hematopoietic MK distribution (bottom). (C) Marker gene expression for MK-LDGs (top) and spatial distribution compared with other LDGs (bottom). (D) Gene ontology (GOBP) and KEGG pathway enrichment for MK-LDG-upregulated genes. (E) Top 20 enriched cell types for MK-LDGs based on WebCSEA analysis. (F) Histone gene expression in MK-LDGs vs. other LDGs. (G) Inferred signaling pathways from MKs to MK-LDGs. (H) Inferred signaling from MK-LDGs to MKs.

Unsupervised UMAP projection revealed the presence of both megakaryocytes (MKs) and low-density granulocytes (LDGs) in the dataset (Fig. 6A). MKs were further classified into two transcriptionally distinct subpopulations: immune MKs and hematopoietic MKs, based on lineage and inflammatory gene expression (Fig. 6B). Similarly, the LDG population was subdivided into MK-LDGs and conventional LDGs using key megakaryocyte-associated markers, such as *PF4* and *PPBP* (Fig. 6C).

To assess the biological relevance of MK-LDGs, we performed functional enrichment analysis on genes upregulated in MK-LDGs relative to other LDGs. Consistent with findings from the discovery cohort, enrichment was observed in pathways associated with platelet activation, innate immune responses, and neutrophil extracellular trap (NET) formation, suggesting conserved functional programs across cohorts (Fig. 6D). Cell-type enrichment analysis using WebCSEA further confirmed the hybrid identity of MK-LDGs, showing strong enrichment for platelet- and megakaryocyte-like signatures (Fig. 6E).

In addition, MK-LDGs displayed high expression of histone-related genes (Fig. 6F), further supporting their potential involvement in NET formation, a key feature of AD pathogenesis.

We also performed CellChat analysis to map intercellular communication within this validation cohort. Notably, active signaling between MK-LDGs and hematopoietic MKs was observed, particularly involving the ITGB2-ICAM2 ligand-receptor pair, which plays a critical role in immune cell adhesion and migration (Fig. 6G and H). This interaction pattern mirrored that of the discovery cohort, suggesting a conserved mechanism of crosstalk between MK and MK-LDG that may contribute to inflammatory amplification in ADs.

Collectively, these validation results substantiate the transcriptional, functional, and intercellular features of MK-LDGs identified in the discovery cohort, highlighting their potential as key immune effectors in systemic autoimmunity.

### 3.7 NET formation in ADs and its correlation with MK-LDG subpopulation

**Fig. 7:**
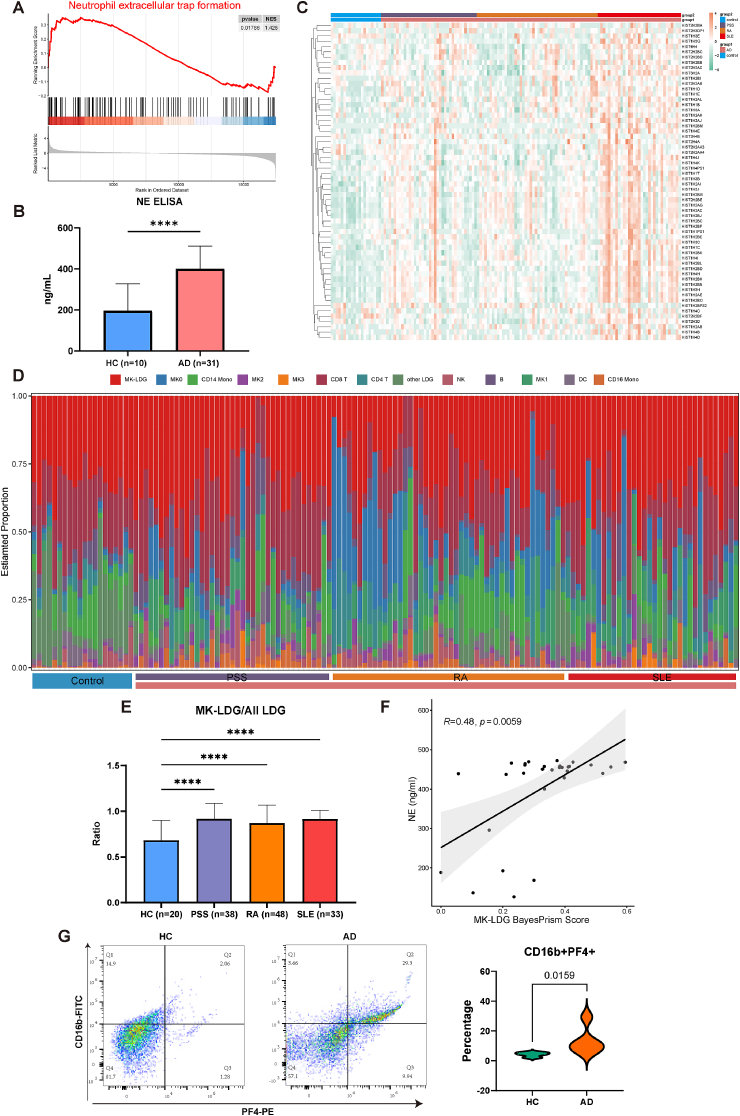
**Neutrophil Extracellular Trap Formation in Autoimmune Diseases.** (A) GSEA plot showing the activation of neutrophil extracellular trap formation. (B) Histogram of neutrophil elastase (NE) concentrations in plasma from healthy controls (HCs) and patients with ADs by ELISA. (C) Heatmap showing the expression levels of histones in AD patients and HCs. (D) Stacked plot showing the proportion of cell types from single-cell data for individual samples. (E) Histogram of the MK-LDG proportions of LDGs in HCs, pSS patients, RA patients, and SLE patients. (F) Scatterplot of the correlation between MK-LDG BayesPrism scores and NE concentrations. (G) Representative flow cytometry pseudocolor plots (left) and violin plot quantification (right) of CD16b + PF4+ MK-LDGs in PBMCs from HCs (n = 4) and AD patients (n = 5). Numbers in the quadrants represent the percentage of cells. The frequency of MK-LDGs is expressed as a percentage of total PBMCs.

To validate the scRNA-seq-derived signatures of MK-LDG–driven NET formation, we analyzed a bulk RNA-seq cohort comprising 119 AD patients (48 RA, 38 pSS, and 33 SLE) and 20 healthy controls (HCs). Gene set enrichment analysis (GSEA) revealed significant activation of NET formation pathways in AD patients (*p* = 0.01786, NES = 1.428, Fig. 7A), indicating a broad activation of NET-related transcriptional programs in systemic autoimmunity. To corroborate these findings at the protein level, ELISA assays demonstrated significantly elevated plasma neutrophil elastase (NE) concentrations in AD patients relative to HCs (p < 0.0001, Fig. 7B). Additionally, heatmap visualization revealed markedly higher histone gene expression in the AD cohort (Fig. 7C), further supporting the occurrence of pathological NETosis.

To assess the association between NET formation and MK-LDG abundance, we performed BayesPrism deconvolution on the bulk RNA-seq data using our scRNA-seq reference (Fig. 7D). This analysis revealed a significantly increased proportion of MK-LDGs within the total LDG pool across all three AD subtypes (*p* < 0.0001, Fig. 7E), underscoring the consistent expansion of this subpopulation in autoimmune settings. Notably, correlation analysis identified a moderate but significant positive association between the inferred MK-LDG abundance and plasma NE concentrations (R = 0.48, *p* = 0.0059, Fig. 7F), suggesting that MK-LDGs may contribute functionally to NET formation in AD. To confirm the protein-level expression of the MK-LDG signature, we performed flow cytometry targeting CD16b and PF4. We successfully identified a distinct population of CD16b^+^PF4^+^ cells within the PBMC fraction, providing direct cellular evidence for megakaryocyte-like granulocytes. Quantitative analysis showed that the proportion of CD16b^+^PF4^+^ MK-LDGs was significantly higher in AD patients (n = 5) than in HCs (n = 3) (*p* < 0.05, Fig. 7G). This protein-level expansion is highly consistent with our scRNA-seq and BayesPrism results, further validating the robust presence of this hybrid population in systemic autoimmunity.

## 4 Discussion

The pathogenesis of autoimmune diseases (ADs) such as pSS, RA, and SLE are characterized by complex immune dysregulation involving both the innate and adaptive arms of immunity. Among innate immune cells, low-density granulocytes (LDGs) have emerged as a pivotal pathological population. Previous studies have predominantly characterized LDGs into mature and immature subsets, noting their association with excessive neutrophil extracellular trap (NET) formation, autoantigen exposure, and tissue injury in SLE([5], [[33], [34], [35]]).

Here, expanding upon this established framework, our study identifies a previously unrecognized subpopulation of LDGs that aberrantly expresses megakaryocyte- and platelet-related genes, particularly *PF4* (*CXCL4*) and *PPBP* (*CXCL7*). These *PF4*^*+*^*/PPBP*^*+*^ MK-LDGsrepresent a specific subset of LDGs that have ectopically acquired megakaryocytic signatures, rather than representing bona fide megakaryocytes. Significantly enriched in AD patients, these cells displayed heightened transcriptional activity associated with NETosis, histone signaling, and immune activation. Crucially, while these findings were initially derived from single-cell transcriptomics, we have substantiated this hybrid population at the protein level. Using flow cytometry in an independent cohort, we confirmed the expansion of CD16b^+^PF4^+^ cells in the peripheral blood of AD patients (n = 5 vs. n = 4), providing definitive cellular evidence that MK-LDGs are a biologically distinct population rather than a computational artifact. This finding suggests that ectopic expression of platelet lineage genes in LDGs may serve as a hallmark of dysfunctional granulopoiesis or aberrant immune programming in autoimmune contexts.

Notably, PF4 and PPBP are canonical markers of megakaryocytes (MKs) and platelets, mediating thrombopoiesis, inflammation, and chemotaxis. Previous studies have highlighted PF4 (CXCL4) as a major autoantigen and a prominent biomarker for disease severity in conditions like systemic sclerosis and SLE, traditionally attributing its source to plasmacytoid dendritic cells and platelets [36]. Their ectopic expression in LDGs raises intriguing questions about granulocyte lineage plasticity in ADs. Our data suggest that these MK-LDGs constitute a transcriptionally distinct population shaped by the inflammatory cytokine milieu or abnormal myelopoiesis, rather than being a product of direct cell-in-cell events. By retaining neutrophil identity while aberrantly expressing MK/platelet-associated genes, these cells may function as duel inflammatory effectors. They possess signaling properties that drive both NET formation and pro-thrombotic signaling, thereby providing a novel cellular link between systemic inflammation and the vascular pathology commonly reported in AD literature [37].

Further supporting their functional relevance, these MK-LDGs co-expressed key histone genes and neutrophil proteases, indicating a primed NETotic state. Given that NETs expose nuclear antigens and histones that contribute to ANA production and immune complex deposition, these aberrant LDGs likely play a central role in perpetuating chronic inflammation, autoimmunity, and end-organ damage in ADs [33]. Simultaneously, elevated expression of pro-platelet factors may facilitate interactions with vascular endothelium and platelets, exacerbating microvascular dysfunction and thrombosis—features increasingly recognized in SLE and related diseases [38].

Mechanistically, our CellChat analysis identified enriched interactions between LDGs and MK subsets, especially involving the ITGB2-ICAM2 and APP-CD74 pathways, which are implicated in immune adhesion, antigen processing, and inflammation [39,40]. These findings suggest a functionally coordinated immune axis between LDGs and MKs that reinforces pro-inflammatory circuits. Of particular interest, the MK2 and MK3 subclusters—enriched in histone and cell-cycle programs—exhibited the strongest transcriptional linkage with MK-LDGs, hinting at shared developmental cues within the inflammatory niche.

Clinically, the emergence of MK-LDGs may represent a biomarker of heightened immune activation, as their abundance correlates with states of excessive NETosis. Therapeutically, targeting the signaling pathways or epigenetic regulators that drive PF4/PPBP expression in LDGs could mitigate both NET-driven inflammation and thromboinflammatory complications, offering a novel precision medicine strategy. This strategy holds promise for precision medicine, particularly for young AD patients for whom long-term immunosuppression carries significant morbidity [40].

Nevertheless, this study has several limitations. First, although we defined MK-LDGs based on scRNA-seq data and validated them via flow cytometry, their exact origin and fate remain unclear. Whether these cells arise from dysregulated granulopoiesis, peripheral activation, or inflammatory transcriptional reprogramming remains to be determined. Second, while our protein-level evidence and transcriptional signatures strongly link these cells to NET formation and autoantigen exposure, direct downstream functional validation in vitro or in vivo remains necessary to fully elucidate their mechanistic contribution to pathogenesis. Finally, although emperipolesis may still occur in ADs, our current dataset does not provide definitive molecular evidence for this process, and thus we avoid mechanistic conclusions on this front. To guide future research, MK-LDGs could be isolated via fluorescence-activated cell sorting (FACS) for ex vivo assays to evaluate their NETosis capacity, while co-culture systems would be instrumental in verifying the predicted ITGB2-ICAM2 and APP-CD74 signaling axes.

In summary, our study reveals a previously unrecognized transcriptional and phenotypic subtype of LDGs characterized by ectopic expression of megakaryocyte-related genes, with potential contributions to NETosis, inflammation, and vascular pathology in autoimmune diseases. These findings offer new insights into the cellular complexity of innate immunity in ADs and highlight *PF4*^*+*^*/PPBP*^*+*^ MK-LDGs as potential biomarkers and therapeutic targets for future translational research.

## 5 Conclusion

This study identifies a novel *PF4*^*+*^*/PPBP*^*+*^ megakaryocyte-like granulocyte (MK-LDG) population that is significantly expanded in autoimmune diseases. While our findings are primarily derived from transcriptomic analysis and computational inference, the existence of this hybrid population is substantiated by protein-level validation via flow cytometry. These MK-LDGs exhibit distinct transcriptional signatures associated with heightened NET formation and dysregulated communication with megakaryocytes through the ITGB2-ICAM2, APP-CD74, and ITGB2-CD226 axes. Although further mechanistic studies are required to fully elucidate their biological roles, our results provide a new framework for understanding innate immune dysregulation and suggest potential therapeutic targets for systemic autoimmunity.

## Ethics approval and consent to participate

All samples were collected in accordance with the ethical requirements and regulations of the Ethics Committee of Shantou Central Hospital. Informed consent was obtained from all the subjects and the studies were conducted under approval (approval number:❲2022❳KY-006).

## Consent for publication

Not applicable.

## Declaration of generative AI in scientific writing

OpenAI ChatGPT 5.2 was used to improve the clarity and grammar of the English writing in the abstract and cover letter. All scientific content and conclusions were generated and verified solely by the authors.

## Funding

The study was supported by 2023 China Ultrasonographers, Rising Stars of Science and Technology Research Program(Class A Grant); The National 10.13039/501100001809Natural Science Foundation of China (No. 82271853); Guangdong Provincial Government-Enterprise Joint Fund (No. 2024A1515220034).

## CRediT authorship contribution statement

**Shaoqi Chen:** Conceptualization, Funding acquisition, Writing – original draft. **Yu Fan:** Conceptualization, Data curation, Formal analysis, Funding acquisition, Investigation, Writing – original draft, Writing – review & editing. **Miaotong Su:** Investigation. **Yuqing Lin:** Investigation. **Shaoyu Zheng:** Investigation. **Zexuan Zhou:** Investigation. **Weijin Zhang:** Investigation. **Jianqun Lin:** Investigation. **Shijian Hu:** Investigation. **Marco Matucci-Cerinic:** Formal analysis, Writing – review & editing. **Daniel E. Furst:** Formal analysis, Writing – review & editing. **Guohong Zhang:** Conceptualization, Methodology, Writing – original draft, Writing – review & editing. **Yukai Wang:** Conceptualization, Funding acquisition, Resources, Writing – review & editing.

## Declaration of competing interest

The authors declare that they have no known competing financial interests or personal relationships that could have appeared to influence the work reported in this paper.

## Data Availability

All sequencing data have been deposited in public, open access repository of the Genome Sequence Archive for Human (https://ngdc.cncb.ac.cn/gsa-human/).

## Appendix A Supplementary data

UMAP projections of PBMC subpopulations across healthy controls and autoimmune disease subtypes.

Multimedia component 1

Demographic and clinical characteristics of the study participants.
