# Supplementary figures and images for: Integrated single-cell transcriptomic analysis identifies PF4+/PPBP+ megakaryocyte-like granulocytes associated with immune dysregulation in autoimmune diseases
Source: Biochem Biophys Rep. 2026 May 10;46:102615. doi: 10.1016/j.bbrep.2026.102615 (PMC13191102; doi:10.1016/j.bbrep.2026.102615)

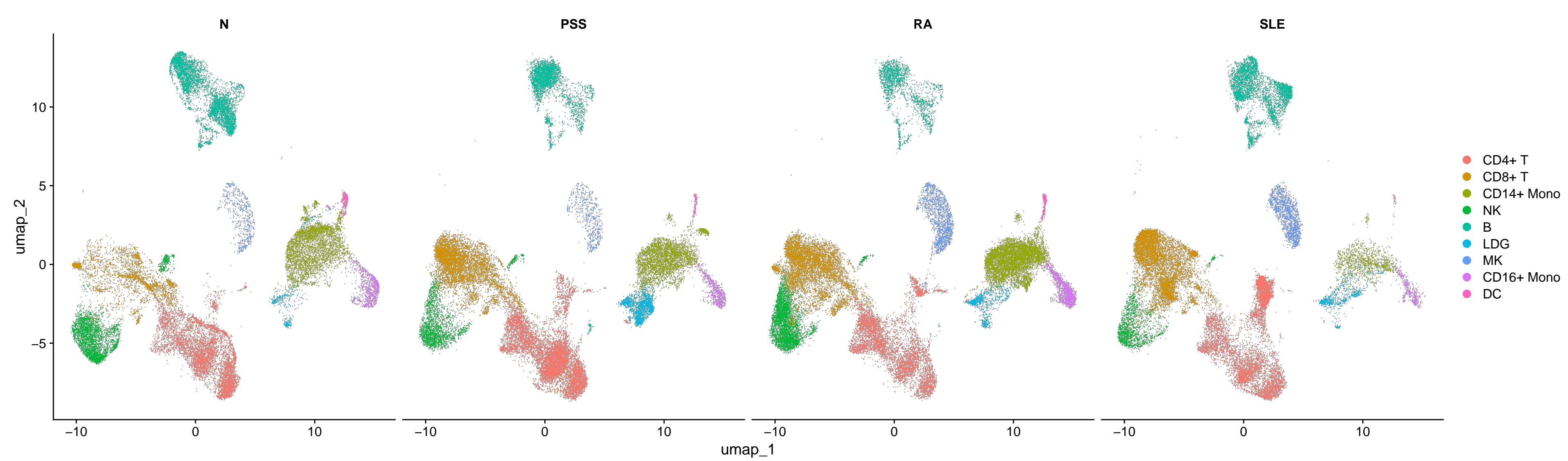

Supplement: Multimedia component 1 [file mmc1.pdf]
